# O6-Methylguanine-DNA methyltransferase protein expression by immunohistochemistry in brain and non-brain systemic tumours: systematic review and meta-analysis of correlation with methylation-specific polymerase chain reaction

**DOI:** 10.1186/1471-2407-11-35

**Published:** 2011-01-26

**Authors:** Marta Brell, Javier Ibáñez, Avelina Tortosa

**Affiliations:** 1 Department of Neurosurgery, Son Dureta University Hospital, Palma de Mallorca, Spain; 2 Department of Basic Nursing, IDIBELL-Universitat de Barcelona, L'Hospitalet de Llobregat, Spain

## Abstract

**Background:**

The DNA repair protein O^6^-Methylguanine-DNA methyltransferase (MGMT) confers resistance to alkylating agents. Several methods have been applied to its analysis, with methylation-specific polymerase chain reaction (MSP) the most commonly used for promoter methylation study, while immunohistochemistry (IHC) has become the most frequently used for the detection of MGMT protein expression. Agreement on the best and most reliable technique for evaluating MGMT status remains unsettled. The aim of this study was to perform a systematic review and meta-analysis of the correlation between IHC and MSP.

**Methods:**

A computer-aided search of MEDLINE (1950-October 2009), EBSCO (1966-October 2009) and EMBASE (1974-October 2009) was performed for relevant publications. Studies meeting inclusion criteria were those comparing MGMT protein expression by IHC with *MGMT *promoter methylation by MSP in the same cohort of patients. Methodological quality was assessed by using the QUADAS and STARD instruments. Previously published guidelines were followed for meta-analysis performance.

**Results:**

Of 254 studies identified as eligible for full-text review, 52 (20.5%) met the inclusion criteria. The review showed that results of MGMT protein expression by IHC are not in close agreement with those obtained with MSP. Moreover, type of tumour (primary brain tumour *vs *others) was an independent covariate of accuracy estimates in the meta-regression analysis beyond the *cut-off *value.

**Conclusions:**

Protein expression assessed by IHC alone fails to reflect the promoter methylation status of *MGMT*. Thus, in attempts at clinical diagnosis the two methods seem to select different groups of patients and should not be used interchangeably.

## Background

The cellular protein O^6^-Methylguanine-DNA methyltransferase (MGMT) is a DNA-repair protein that removes mutagenic and cytotoxic adducts from O^6^-guanine in DNA. Alkylating agents are among the most widely used chemotherapeutic drugs in human cancer. Alkylation induced by these compounds can produce either lethal double-strand cross-links, as is the case with bifunctional nitrosoureas (BCNU), or induce mismatch abortive repair and DNA fragmentation, as is the case with temozolomide [1-4]. The toxicity of alkylating agents is reduced in the presence of MGMT. Thus, MGMT confers resistance to alkylating agents in a wide spectrum of human tumours by reversing DNA toxicity. In brain neoplasms, hypermethylation of CpG islands in the *MGMT *gene promoter region, rather than mutation or deletion, is the major mechanism for the loss of MGMT function [2,5-7]. As a consequence, tumours with epigenetic silencing of *MGMT *gene become more sensitive to the killing effects of alkylating agents. Moreover, several studies have demonstrated that epigenetic silencing of MGMT is a relevant prognostic factor in patients with glioblastoma, anaplastic glioma and low grade glioma [8-14]. In fact, MGMT status has recently been recommended as a stratifying factor for patients in glioma trials [15,16].

Many methods and protocols have been applied for MGMT analysis in gliomas, but to date there is no consensus on which strategy should be primarily employed [17]. Methylation-specific polymerase chain reaction (MSP) is the most commonly used test [9]. Indeed, in glioblastoma clinical trials, a strong correlation of the methylation status of *MGMT *with temozolomide response and patient outcome was shown. However, there are some methodological problems that limit the usefulness of this method in a routine diagnostic setting: it is complex, time-consuming, and highly dependent on tissue quality [18,19]. MGMT status can also be assessed by analyzing protein expression by immunohistochemistry (IHC). IHC is a reliable, commonly used method in diagnostic histopathology that is available in most laboratories. In addition, IHC is easier to use, less expensive and faster than MSP [20-29], and consequently it has become the most frequently used method for the detection of MGMT protein expression in the past decade [30]. In this line, some retrospective clinical reports have also shown a prognostic association between MGMT protein expression and/or activity and outcome.

However, studies aimed at evaluating the correlation between aberrant promoter methylation and loss of protein expression have yielded contradictory results, not only in brain tumours but also in other neoplasms. While we and other authors have shown that the relationship between *MGMT *promoter methylation status and MGMT protein expression is not absolute [31], other studies have found a strong correlation between homogeneous immunoreactivity and unmethylated promoter [32]. At present, there is a lack of data on which to base recommendations for a specific method or protocol for MGMT testing. Accordingly, there is a strong need for systematic comparisons and validation of intra- and interlaboratory reproducibility of different methods for MGMT assessment in order to identify the best method for clinical MGMT testing [33].

The aim of this study was to perform a systematic review and a meta-analysis of the correlation between MGMT IHC and MSP in a large array of human brain and non-brain systemic tumours. Our primary objective was to assess the diagnostic accuracy of IHC at different *cut-off *values for test positivity. Because test accuracy is not a fixed property of a test [34], we have also studied several possible sources of heterogeneity such as subgroups of patients, differing interpretations of results, and study design features.

## Methods

This systematic review and meta-analysis was performed following previously published guidelines [34-37].

### Literature Search

A computer-aided search of MEDLINE (1950-October 2009), EBSCO (1966-October 2009) and EMBASE (1974-October 2009) was performed for relevant publications. Medical Subject Heading (MeSH) terms with accompanying entry terms were used (Additional file 1). To identify additional published, unpublished and ongoing studies, we entered relevant studies identified from the above sources into PubMed and then used the Related Articles function. The Science Citation Index was searched to identify articles citing relevant publications. The reference lists of all selected papers were also reviewed for search completion. Only English-language literature was considered eligible. Titles and abstracts were screened by two reviewers (M.B. and J.I.) to identify relevant articles. Discrepancies were resolved by consensus.

### Criteria for inclusion of studies

Studies meeting inclusion criteria were those comparing MGMT protein expression by IHC with *MGMT *promoter methylation by MSP as the reference test in the same cohort of patients. Not only brain tumour series but also others involving any type of cancer were considered eligible whenever both diagnostic tests were used in the same population. Studies on cellular lines were excluded. Information had to be available to allow the construction of the diagnostic two-by-two table with its four cells: true positive, false negative, false positive and true negative.

### Index test and reference test

IHC performed with different commercially available antibodies was the test under evaluation and MSP was considered the reference test, as it is the most commonly used.

### Quality assessment and data extraction

Methodological quality of included studies was assessed independently by two observers (M.B. and J.I.) using the QUADAS tool [38] which was specifically developed for systematic reviews of diagnostic test accuracy studies. The tool is based on 14 items scored as "yes", "no", or "unclear". The items from the QUADAS tool and their interpretation can be found in Additional file 2.

Data extraction was performed independently by two authors (M.B. and J.I.), and included author and date, journal of publication, time of data collection, testing procedure, study population, reference test, performance of the reference test and of the index test, *cut-off *value used for immunolabeling, QUADAS-items, whether histological analysis of the tissue used for DNA extraction was performed or not, the percentage of methylated cases by MSP, the effect of methylated promoter/protein expression on survival, and data for two-by-two table. A quality score was not used as a weighting variable because of its subjectivity [39]. The STARD [40] checklist and flow diagram were also followed as recommended.

### Data analysis

Studies reporting insufficient data for the construction of a two-by-two table were excluded from final analyses. Data from the two-by-two tables were used to calculate sensitivity, specificity and diagnostic odds ratio for each study. We present individual study results graphically by plotting the estimates of sensitivity and specificity (and their 95%CI) in both forest plots and the receiver operating characteristic (ROC) space. Heterogeneity was investigated in the first instance through visual inspection of the relationship between pairs of accuracy estimates in forest plots and sROC space [41]. As one of the primary causes of heterogeneity in test accuracy studies is the threshold effect, which arises when different *cut-offs *are used in different studies to define a positive (or negative) test result, the computation of the Spearman correlation coefficient between the logit of sensitivity and logit of 1-specificity was also performed. A strong positive correlation suggests this threshold effect. In order to explore for heterogeneity other than threshold effect, the chi-square and Cochrane-Q tests were used. A low p-value suggests the presence of heterogeneity beyond what could be expected by chance alone. The inconsistency index (I-squared) was used to quantify the amount of consistency--that is, the percentage of total variation across studies due to heterogeneity rather than chance. Statistical heterogeneity can be defined as low, moderate and high for *I^2 ^*values of 25%, 50% and 75% [42]. When a substantial heterogeneity was found, the reasons for it were explored by relating study level covariates to diagnostic odds ratio, using meta-regression techniques. Subgroup analyses trying to identify homogeneity were then performed but in all cases pooling was done using methods based on a random effect model. This model assumes that in addition to the presence of random error, differences between studies can also result from real differences between study populations and procedures, and it includes both within-study and between-study variations. Sensitivity and specificity were compared between these subgroups using the z-test [36]. Publication bias was examined by construction of a funnel-plot. The x-axis consisted of the natural logarithm of the diagnostic odds radio, and the y-axis was the standard error, which is considered the best choice [43]. In the absence of bias the graph resembles a symmetrical inverted funnel because the accuracy estimates from smaller studies scatter more widely at the bottom of the graph, with the spread narrowing with increasing accuracy among larger studies. If there is publication bias the funnel plot will appear skewed and asymmetrical. Although useful, interpretation of the funnel-plot is subjective; for this reason the Egger's regression test became necessary in order to measure the funnel-plot asymmetry numerically [44]. The intercept provides a measure of the assymetry: the greater its deviation from zero the more pronounced the asymmetry.

Statistical analysis was performed using Meta-Disc software http://www.hrc.es/investigacion/metadisc_en.htm[45]. The analysis for publication bias was performed using CMA-1 http://www.Meta-Analysis.com. Two-sided *P *< .05 was considered to be statistically significant.

## Results

### Results of the search and study characteristics

**Figure 1:**
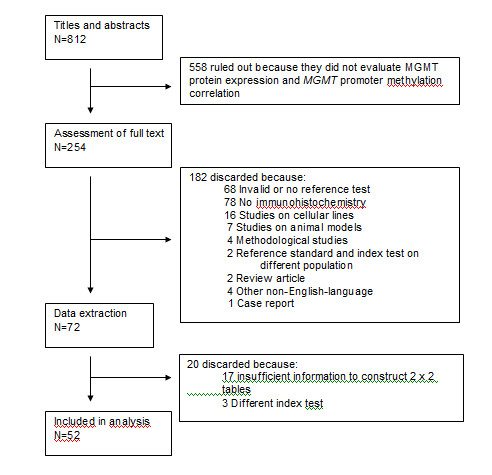
**Flow diagram of inclusion process**.

The initial search strategy yielded 812 articles, 254 of which were eligible for full-text review. Of these, 182 studies were ruled out, and 72 were selected for data extraction. All selected studies were diagnostic cohort studies. Seventeen studies [20,26,30,46-59] reported data that were insufficient for the construction of the two-by-two table, and in 3 studies [60-62] protein expression was assessed by a test other than IHC. These 20 studies were not included in the analysis. Thus, 52 relevant studies constitute the basis of this analysis (17 glioma studies, 3 non-glioma brain tumour studies and 32 non-brain systemic tumour studies) comprising a total of 2,943 patients: 539 with primary brain tumours, 178 with brain metastases of various solid tumours and 2,226 with non-brain systemic cancer (Figure 1). Additional file 3 and Additional file 4 show the characteristics of included studies.

Regarding the IHC analysis, the most commonly used antibody was anti-MGMT mouse monoclonal clone MT3.1 (from Dako, Chemicon International, NeoMarkers, Santa Cruz Biotechnology or Kamiya Biomedical Laboratories), which was reported in 39 out of 52 (75%) studies, followed by anti-MGMT mouse monoclonal antibody clone MT23.2 (from Zymed Laboratory) which was used in 4 (7.6%) series. Other commercially available anti-MGMT antibodies were reported in 7 (13.4%) additional studies. In one study, no laboratory specification was reported [63]. MGMT immunoexpression was qualitatively analyzed in 16 out of 52 (30.8%) studies. Accordingly, a semiquantitative score which estimates the fraction of positive cells was used in 36 studies (69.2%). Indeed, MGMT expression was evaluated by semiquantitative scoring in the majority of the brain tumour studies (18 out of 20) and in 18 out of 32 systemic tumour series. As shown in Additional file 3 and Additional file 4, different *cut-off *values were used, ranging from 5% to 80%. Statistically significant association between IHC and MSP was found in 9 out of 20 brain tumour studies, while in the group of non-brain systemic tumours this concordance between the two tests was observed in 29 of the 32 series (90.6%).

Regarding the MSP analysis, genomic DNA was isolated from formalin-fixed paraffin-embedded tissue in 26 studies (50%), whereas in 21 cases it was isolated from fresh-frozen samples (40.3%). In five studies (9.6%) DNA was isolated from both types of specimens. Sodium bisulfite modification of isolated DNA was performed using commercially available DNA methylation kits in nearly half of them (24 out of 52) including DNA Methylation Kit (Zymo Research), Methylamp DNA Modification Kit (Epigentek Inc), CpGenome DNA Modification Kit (Intergen), and Fast DNA Modification Kit (Chemicon).

### Methodological quality of included studies

**Figure 2:**
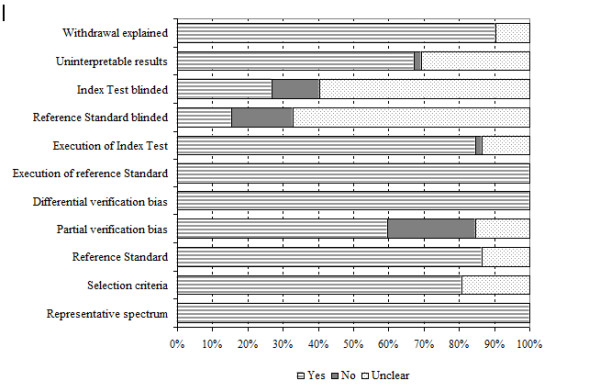
**Methodological quality graph**.

Figure 2 and Additional file 5 show assessment of methodological quality of included studies using the QUADAS tool. Inclusion of a representative patient spectrum and explanation of selection criteria or withdrawals did not constitute a limitation of any study. Eight studies reported the use of some modification of the original MSP as the reference test [32,64-70]. In approximately one quarter of the studies, partial verification bias was not clearly avoided as not all cases evaluated with the index test were verified using the reference test. Some authors reported that only tumour samples with an estimated tumour cell content of at least 80% were used for molecular studies [71], while in others this requirement was not clearly reported.

Immunohistochemical expression was scored semiquantitatively or qualitatively in all but six studies [1,64,69,72-74], in which interpretation of the index test was not satisfactorily explained by the authors. We did not expect any differential verification bias because all studies used the same reference test for the whole cohort of patients. In 84.6% of the studies, the authors did not unequivocally state whether assessment of the reference test was blinded for the IHC results, and in 73% of the series, no details were reported about blinding of the index test. Seventeen studies reported no details about any uninterpretable or indeterminate index test results [2,64,66,70,73-85].

### Data analysis

**Figure 3:**
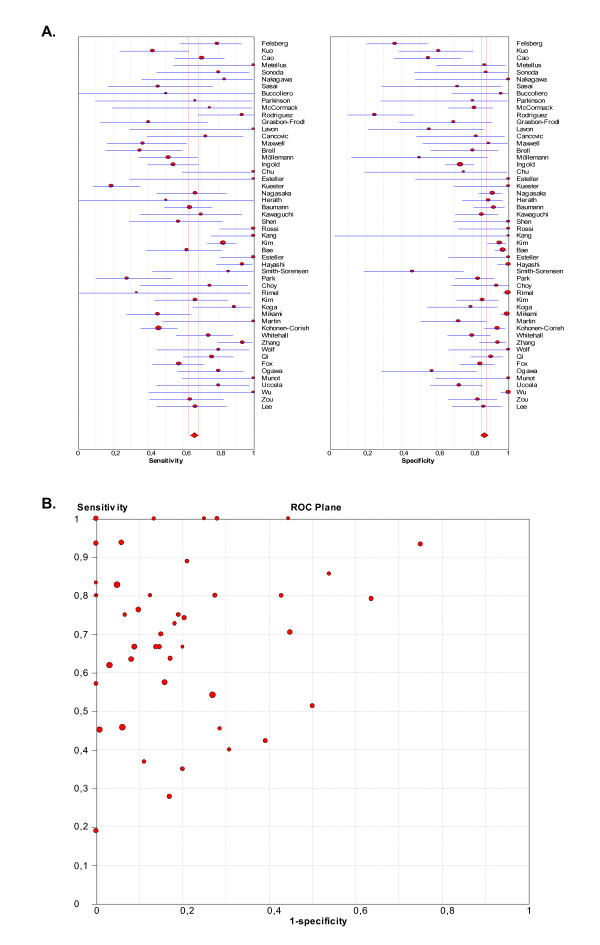
**Forest-plots for sensitivity and specificity and ROC Space representation from all elegible studies**. (A) Forest-plots for sensitivity and specificity with corresponding 95% CI. (B) ROC Space representation of sensitivity against (1-specificity) for each study.

Tabular results for sensitivity, specificity, likelihood ratios and diagnostic odds ratios for all studies are given in Additional file 6. At this early stage of the analysis, the pooled summary of accuracy measures was not taken into account, as significant heterogeneity was suggested when observing the forest plots and the sROC space (Figures 3A and 3B). No statistically significant difference was observed when exploring for threshold effect, either considering all studies (n = 52, Spearman correlation coefficient = -0.022; p = 0.881) or just the subgroup of studies in which semiquantitative scoring was used (n = 36, Spearman correlation coefficient = 0.037; p = 0.833). However, statistical heterogeneity was observed for sensitivity (chi-square = 234.28; df = 42 (p < 0.0001), inconsistency (I^2^) = 79.5%), specificity (chi-square = 300.84; df = 48 (p < 0.0001), I^2 ^= 84%), positive LR (Cochrane-Q = 265.33; df = 48 (p < 0.0001), I^2 ^= 81.9%), negative LR (Cochrane- Q = 201.46; df = 48 (p < 0.0001), I^2 ^= 76.2%), and diagnostic odds ratio (Cochrane-Q = 143.88; df = 48 (p < 0.0001), I^2 ^= 66.6%), thus suggesting other sources of heterogeneity across the studies. Accordingly, meta-regression analysis with the following covariates was performed: 1) type of tissue used for MSP, as paraffin embedded specimens may not yield enough quality DNA to successfully perform the test [86]; 2) anti-MGMT antibody used, as the best agreement between MSP and IHC results seems to be achieved when using the MT23.2 antibody [33]; and 3) type of tumour analyzed. Results suggest that the type of tumour is strongly associated with accuracy (RDOR 5.36; 95% CI[2.42-11.86], p < 0.01) (Additional file 7).

In the next step, a second meta-regression analysis was performed for the subgroup of 36 studies in which semiquantitative scoring for IHC was used, and the *cut-off *value was also included as covariate. Interestingly, the type of tumour (primary brain tumour *vs*. others) was also selected as an independent covariate of accuracy estimates beyond *cut-off *value, type of tissue or type of antibody used. MGMT protein expression by IHC for brain tumours is associated with a more than four-fold lower accuracy compared to other tumours (RDOR 4.38; 95% CI[1.82-10.54], p = 0.0017) (Additional file 8).

The final step of the analysis was pooling accuracy estimates in homogeneous subgroups of studies with identical type of tumour and identical *cut-off *value. To rule out an implicit threshold effect due to naturally occurring variations in the interpretation between observers, laboratories or devices, the Spearman rank correlation was performed, and no evidence of threshold effect within these groups was found. Overall, the summary estimates found ranged from 0.53; 95% CI[0.33-0.73] to 0.79; 95% CI[0.73-0.84] for sensitivity and from 0.60; 95%CI[0.52-0.67] to 0.93; 95%CI[0.91-0.95] for specificity (Additional file 9).

**Figure 4:**
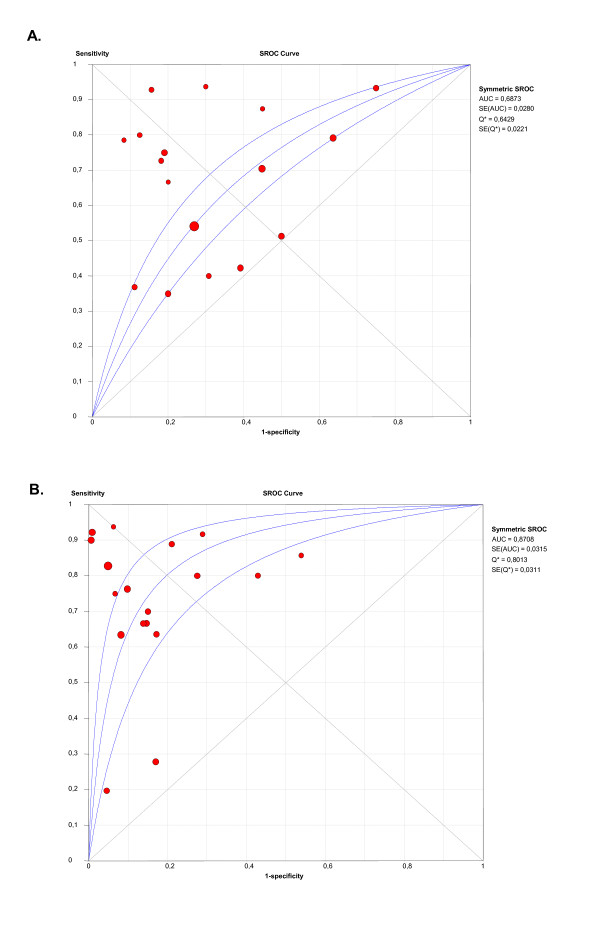
**SROC curves for studies with IHC semiquantitative scoring**. (A) SROC curve for brain tumour studies with IHC semiquantitative scoring. (B) SROC curve for non-brain tumour studies with IHC semiquantitative scoring.

To further evaluate diagnostic accuracy for MGMT protein expression by IHC when identical scoring and *cut-off *values were used, we determined the Q*index. Figures 4A and 4B show that the Q*index was 0.64 and the area under the curve (AUC) 0.68 for brain tumour studies, while the Q*index was 0.80 and the AUC 0.87 for non-brain tumour series, indicating a statistically significant higher level of overall accuracy in systemic tumours (z-statistic 4.354, p < 0.0001). This difference remained statistically significant when we included all studies in the analysis (z-statistic 5.722, p < 0.0001).

**Figure 5:**
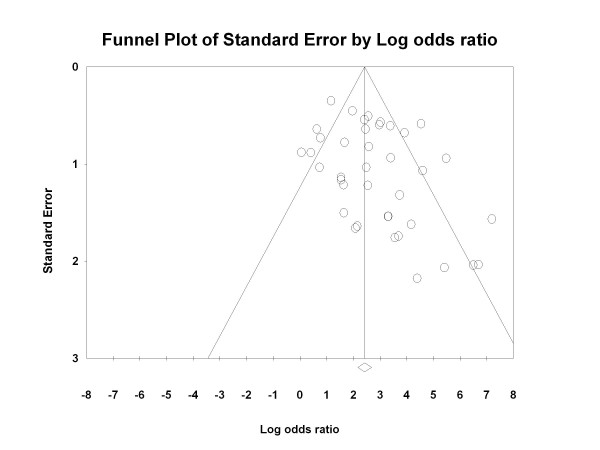
**Funnel-plot for the assessment of potential publication bias**.

Finally, the Egger's regression test for the detection of publication bias showed an asymmetrical distribution of the points in the funnel-plot (Intercept 1.55; 95%CI[0.61-2.49], p = 0.002) (Figure 5), indicating a potential publication bias.

## Discussion

The relevance of MGMT status as a potential prognostic or predictive factor in malignant glioma patients is supported by a number of independent studies. At present, detection of *MGMT *promoter methylation by MSP is the most commonly used method and for this reason it is considered the reference test in the present review. However, concerning day-to-day clinical practice, MSP is not yet part of the routine diagnostic work-up while MGMT assessment at RNA or protein-level are used [22,33]. The exact incidence of promoter methylation, protein or RNA expression varies according to the assessment test and among different studies [87]. An optimal method for diagnostic purposes should be widely available, easy to establish, cost-effective, reproducible both within a given laboratory and among different laboratories, and capable of yielding results that show consistent association with patient outcome [19,33]. In this regard MSP is a highly sensitive qualitative technique, but IHC has several advantages over it [88].

Although strong agreement between MSP and IHC has been previously reported, there is growing evidence that *MGMT *promoter methylation assessment through MSP does not correlate well with MGMT protein expression as detected by IHC in brain tumours [25,26,31,68,89]. In addition, some studies have shown that *MGMT *promoter methylation and MGMT protein expression cannot be used interchangeably to predict patient survival or glioma chemosensitivity [68,90]. Results from the present meta-analysis support this evidence and suggest that cases selected by IHC may not always correspond to those selected by MSP. In fact, diagnostic accuracy estimates for MGMT protein expression by IHC were significantly lower for brain tumours than for other non-brain tumours (sensitivity, 53-64% vs. 60-81% respectively; specificity, 60-84% vs. 80-93% respectively). Similarly, positive and negative likelihood ratios did not provide convincing diagnostic accuracy for IHC in brain tumours (Additional file 9). Accordingly, the type of tumour (primary brain *vs*. non-brain systemic tumour) turned out to be an independent covariate of accuracy estimates in the meta-regression analysis beyond other methodological covariates such as *cut-off *value and type of antibody.

The reasons for these findings are not clear and different putative causes must be taken into consideration. First, there is a lack of a consistently defined *cut-off *value for the semiquantitative immunohistochemical scoring. Capper et al. proposed a *cut-off *of 15% immunolabeled cells for GBM and 35% for low grade gliomas [22], Nakasu et al. proposed a *cut-off *value of 10-20% [88], and Preusser et al. found the best agreement between MSP and IHC results when using a *cut-off *of 50% [20]. It is important to note that the *cut-off *value was not an independent covariate of accuracy in the present meta-regression analysis, whereas the type of tumour (primary brain *vs *non-brain) was independently associated with greater accuracy (Additional files 7 and 8). In addition, interobserver variability in discriminating positive and negative cells, specific immunostaining and background is another technical aspect of the IHC procedure [20]. Even when studies use the same explicit threshold, their implicit threshold may differ, especially if interpretation of the test requires pathology judgement [35]. Importantly, histological analysis of the tissue used for DNA extraction is not always performed (Additional file 3 and Additional file 4), and when the area of tumour used for MSP analysis is different from the one studied with IHC, necrosis and/or an overlarge sample of normal tissue might hamper the MSP results. Third, due to the fact that MSP relies on the different susceptibility of methylated versus unmethylated cytosines to sodium bisulfite modification and subsequent selective primers amplification, it is highly dependent on tissue quality and quantity, primer design, bisulfite treatment adequacy and PCR conditions [19]. Finally, MSP is so highly sensitive that a methylation band may be obtained even if cells that carry *MGMT *promoter methylation represent a small proportion among the majority of cells with unmethylated promoter [1]. Conversely, IHC may not be able to detect small clusters of cells that have lost protein expression [91].

Apart from these technical issues, there are other confounding factors that may lead to false positive methylation results. Although it has been stated that the presence of a methylated *MGMT *allele can only be attributed to neoplastic cells [8,10,92], some authors have demonstrated that *MGMT *promoter methylation may occur in non-neoplastic central nervous system tissue [3] or in normal-appearing mucosa several centimetres away from digestive tumours [56,93]. Moreover, Candiloro et al. [94] have shown low levels of methylation in peripheral blood of healthy individuals with the T allele of the rs16906252 polymorphism.

Moreover, regulation of MGMT expression in brain tumours seems to be a complex phenomenon in which abnormal methylation of the promoter region may not be the only determining factor [1,47,95-97]. Similar to genetic and chromosomal events, epigenetic changes may also be tissue- and tumour-specific [98,99]. In fact, the inconsistency between promoter methylation and protein expression assessed by IHC in gliomas is not limited to the *MGMT *gene, but has also been observed for other genes such as *PTEN *[100]. Gliomas are heterogeneous tumours and intratumoural heterogeneity of MGMT staining and methylation is a well-known event. Over time, variations in the methylation status of *MGMT *promoter within the same tumour have also been described, although the relevance of these events is unclear [31,89,101]. Interestingly, some factors, such as glucocorticoids, ionizing radiation and chemotherapy, can induce MGMT expression [26,102]. Thus, a further question to be addressed is whether tumour recurrences exhibit the MGMT status as the pre-treatment tumour or a different one. Unfortunately, data on this topic are limited and contradictory [103]. While some studies have demonstrated an increase in MGMT immunostaining [84] or a lower frequency of *MGMT *promoter methylation [87,104,105] in recurrent gliomas after chemotherapy, other authors have not observed any change [84,103,106]. Finally, both an increase and a decrease in MGMT expression have also been described for recurrent tumours [22,76,87,107-109]. A higher protein expression might indicate that the *MGMT *gene has been up-regulated by the treatment, although other possible explanations, such as selection of chemoresistant cells with high MGMT protein levels or intratumoral regional variations, can not be excluded [26,84,109].

Finally, methylation is not biallelic in some tumours, leaving one allele actively expressing the protein while *MGMT *promoter methylation may be also observed [110]. In fact, *MGMT *gene is located on chromosome *10q*, a region lost in the vast majority of GBM, implying that even in those GBM without promoter methylation, *MGMT *haploinsufficiency is likely [101]. Moreover, *MGMT *promoter CpG islands may present a differential pattern of methylation along the region, with some CpGs being more important than others with regard to gene transcription. In this sense, it has been suggested that the region commonly investigated by MSP might not to be among those that best correlate with protein expression [90].

In an attempt to avoid some of the above mentioned problems, quantitative or semiquantitative methods such as MethylLight^® ^quantitative MPS, pyrosequencing, COBRA, etc. [66,67,70,83,87,89,111] have been reported by different groups in recent years. Whether these methods are more appropriate than MSP remains to be demonstrated in large cohorts of patients. Quantitative methods seem to provide better discrimination than classical gel-based MSP. However, as Karayan-Tapon et al. [46] note, before these methods can be used as clinical biomarkers, validation of them is required. Whichever gene is used for normalization, no quantitative-MSP assay can give a real, absolute measurement, and this might be a restriction. Moreover, completely quantitative or semiquantitative assays that normalize to a control gene or the copy number of the unmethylated *MGMT *promoter sequence might underestimate *MGMT *methylation, because contaminating nontumoral tissue will contribute to the signal of the normalizing gene [112].

Both MGMT status at protein level and promoter methylation have been correlated with prognosis and chemosensitivity in glioma patients. As is shown in Additional file 3 and Additional file 4, the prognostic and predictive value of protein expression has been evaluated in some studies with contradictory results. Several authors have reported a significant association of MGMT expression assessed by immunohistochemistry with patients' overall or progression-free survival [22,23,31,88,113-117]. Some of them have even shown MGMT protein expression to be an independent predictor in the multivariate analysis [31,84,85,115,116,118,119], whilst others have demonstrated a lack of correlation [29,46,58,74]. However, most published data were obtained from heterogeneous groups of patients with different grades and histologies, as well as distinct treatment protocols [31]. Although differences in study design could explain, at least in part, these contradictory results, other possibilities should be considered. In this sense, while those neoplastic cells that do not express MGMT may not be able to correct DNA damage induced by chemotherapy, loss of MGMT expression can also be a negative prognostic factor because of an increased susceptibility to acquiring other mutations [120-122]. Furthermore, due to variable interobserver agreement, insufficient correlation with MGMT promoter methylation status and the lack of a firm association with patient outcome [20,29,103], MGMT IHC has not proved to be a clinically usable biomarker for routine diagnostic purposes and clinical decision-making.

Our review has several limitations. First, we excluded 17 studies because they did not provide data allowing construction of two-by-two tables, potentially resulting in less precise estimates of pooled diagnostic accuracy. Second, the statistical power of this meta-analysis was limited by the relatively small sample size of most included studies. Third, the QUADAS tool revealed that in approximately two-thirds of the studies partial verification bias was not clearly avoided, as not all cases evaluated with the index test went on to receive verification using the reference test. Another important aspect of study quality is the blinding of results of experimental and reference tests [123]. Unfortunately, in 84% of the studies, assessment of the reference test blinded for the IHC results was not explicitly stated by the authors, and in 73% of them no details were reported about blinding of the index test. Finally, publication bias was found in the present meta-analysis. Exclusion of non-English-language studies could contribute to explaining this fact, although a preference for publishing studies reporting positive results is a more plausible explanation [44].

## Conclusions

The present systematic review and meta-analysis has shown that assessment of MGMT protein expression by IHC is not in good concordance with results obtained with the MSP test. Discordance between the two tests seems to be higher for brain tumours even when comparing subgroups with identical *cut-off *value. Therefore, it seems that *MGMT *promoter methylation does not always reflect gene expression and, accordingly, the two methods cannot be used interchangeably. We conclude that protein expression assessed by IHC alone fails to reflect the promoter methylation status of *MGMT*, and thus in clinical diagnostic attempts the two methods will not select the same group of patients. This fact can be of crucial importance when stratifying patients in clinical studies according to their MGMT status.

Despite all the above mentioned aspects, MSP currently remains the most established method and the best approach to assessing MGMT status. It is also the technique for which the most convincing clinical correlations have been reported and, thus, it should be considered the reference test. Unfortunately, it is a relatively complex and time-consuming method not apt for routine clinical implementation in many centres [19].

However, the analytical and clinical performance of MGMT immunoassaying seems to be inappropriate for routine diagnostic purposes. This fact, along with the lack of a robust association with *MGMT *promoter methylation as demonstrated in the present meta-analysis, precludes its use as a valuable biomarker for clinical decision making. It remains to be determined whether novel anti-MGMT antibodies directed against other epitopes would improve its performance [20].

Accordingly, some authors have suggested the feasibility of using MSP combined with IHC for prognostic and predictive purposes [104,116]. Immunohistochemistry may represent a useful preliminary test to identify methylated cases while MSP should be performed in non-immunoreactive cases to identify truly methylated tumours [70]. Again, this issue deserves further investigation.

## Competing interests

The authors declare that they have no competing interests.

## Authors' contributions

All authors have participated sufficiently in the work to take public responsibility for appropriate portions of the content. MB and JI have made the design, review of the literature, and acquisition and analysis of data. They have also contributed to manuscript drafting and have approved its final version. AT has been involved in the interpretation of data, manuscript writing and critical revision, and has also approved the final version.

## Pre-publication history

The pre-publication history for this paper can be accessed here:

http://www.biomedcentral.com/1471-2407/11/35/prepub

## Supplementary Material

Additional file 1 **Computer-aided search strategy**. Click here for file

Additional file 2 **QUADAS items and their interpretation **[9,33,124]. Click here for file

Additional file 3 **Characteristics of glioma studies selected for full text review **[2,18,26,29-31,46,47,58,59,68,71,76,84,85,87,89,115,116,118,119,125-127]. Click here for file

Additional file 4 **Additional file 1**. Characteristics of non-glioma studies included in the analysis [1,32,63-67,69,70,72-75,77-83,91,95,96,128-139]. Click here for file

Additional file 5 **Evaluation of quality of the included studies using the QUADAS tool **[1,2,29,31,32,63-85,87,89,91,93,95,96,115,116,118,119,125-133,135-139]. Click here for file

Additional file 6 **Tabular results of Sensitivity, Specificity, Likelihood Ratios, and Diagnostic Odds Ratio **[1,2,29,31,32,63-87,89,91,95,96,115,116,118,119,125-139]. Click here for file

Additional file 7 **Results of meta-regression analysis for all studies**. Click here for file

Additional 8 **Results of meta-regression analysis for the subgroup of studies using semiquantitative scoring for IHC assessment**. Click here for file

Additional file 9 **Summary of results**. Click here for file

## Ackowledgments

We thank G. Frontera of the Investigation Unit, Son Dureta University Hospital, Palma de Mallorca, Spain, for statistical advice, and Tom Yohannan for language assistance.

This study was supported by grants from the Ministerio de Sanidad y Consumo (Fondo de Investigación Sanitaria 08/1085; Instituto de Salud Carlos III-RETIC RD06/0020/0097) and the Fundación Médica Mútua Madrileña, 2007.
