# Supplementary material for: O6-Methylguanine-DNA methyltransferase protein expression by immunohistochemistry in brain and non-brain systemic tumours: systematic review and meta-analysis of correlation with methylation-specific polymerase chain reaction
Source: BMC Cancer. 2011 Jan 26;11:35. doi: 10.1186/1471-2407-11-35 (PMC3039628; doi:10.1186/1471-2407-11-35)
Supplement: Additional file 1 — Computer-aided search strategy. [file 1471-2407-11-35-S1.DOC]

**Additional file 1:** **Computer-assisted search strategy**

| **MEDLINE (through PubMed)** |
| --- |
| - (MGMT[All Fields] OR (O6-Methylguanine[All Fields] AND ("dna"[MeSH Terms] OR "dna"[All Fields]) AND ("methyltransferases"[MeSH Terms] OR "methyltransferases"[All Fields] OR "methyltransferase"[All Fields]))) AND (("immunohistochemistry"[MeSH Terms] OR "immunohistochemistry"[All Fields]) OR ("gene expression"[MeSH Terms] OR ("gene"[All Fields] AND "expression"[All Fields]) OR "gene expression"[All Fields] OR "expression"[All Fields])) |
| **EMBASE** |
| - 'mgmt' AND ('immunohistochemistry'/exp OR 'immunohistochemistry') AND ('expression') |
| **EBSCO** |
| - ((mgmt or O6 METHYLGUANINA DNA METHYLTRANSFERASE) and (EXPRESSION or IMMUNOHISTOCHEMISTRY)).af. |
