# Supplementary material for: O6-Methylguanine-DNA methyltransferase protein expression by immunohistochemistry in brain and non-brain systemic tumours: systematic review and meta-analysis of correlation with methylation-specific polymerase chain reaction
Source: BMC Cancer. 2011 Jan 26;11:35. doi: 10.1186/1471-2407-11-35 (PMC3039628; doi:10.1186/1471-2407-11-35)
Supplement: Additional file 2 — QUADAS items and their interpretation [9,33,124]. [file 1471-2407-11-35-S2.DOC]

**Additional file 2: QUADAS items and their interpretation**

1. Representative spectrum?

Representative patient spectrum was defined as any cohort of patients with either brain tumors or other types of cancer in whom correlation between MGMT expression by IHC and *MGMT* promoter methylation analysis by MSP could be clearly evaluated.

2. Clear description of selection criteria?

Selection criteria were evaluated for all studies and scored as clearly described if, minimally, it was stated in which department the participants were recruited, as previously described [124].

3. Acceptable reference standard test?

As there are, so far, limited data on the feasibility for use in the clinical setting of other DNA-based methods different from MSP [33], the corresponding item was scored as “unclear” when any modification of the original MSP was used.

4. Any delay between tests?

Only the results of the index test and the reference standard collected on the same tumor at the same time were included for the analysis.

5. Partial verification bias avoided?

Partial verification bias occurs when not all the study group receives confirmation by the reference standard. If it was clear from the study that all cases evaluated with the index test went on to receive verification using the reference test, this item was scored as “yes”.

6. Differential verification bias prevented?

If the reference test was the same for the entire study group this item was scored “yes”.

7. Was the reference standard independent of the index test?

We did not assess this item as the index test does not form part of the reference standard.

8. and 9. Were the execution of the index test and the reference standard described in sufficient detail to permit their replication?

Not only did we assess whether the index test and the reference test were clearly described, but also the type of commercial antibody used, the *cut-off* value for ICH, and whether sodium bisulphite was modified with the aid of commercially available kits.

10. and 11. Were the index test results interpreted without knowledge of the results of the reference standard and vice versa?

These items were scored “yes” if the authors stated explicitly that the assessment of the index test was blinded for the MSP results and vice versa.

12. Were the same clinical data available when the test results were interpreted as would be the case in clinical practice?

We did not use this item because the availability of clinical data does not affect the interpretation of the index test.

13. Uninterpretable/intermediate test results reported?

It is well known that a relatively high percentage of paraffin embedded samples do not yield DNA of sufficiently high quality to successfully perform MSP. Moreover, in the study by Hegi et al. [9] *MGMT* methylation status could only be determined in 67% of the tissue specimens. This item was scored as “yes” if the total of cases that could be analyzed and the inconclusive results were reported.

14. Withdrawals from the study explained?

In case there were withdrawals it was indicated whether or not these were explained.
