# Supplementary material for: O6-Methylguanine-DNA methyltransferase protein expression by immunohistochemistry in brain and non-brain systemic tumours: systematic review and meta-analysis of correlation with methylation-specific polymerase chain reaction
Source: BMC Cancer. 2011 Jan 26;11:35. doi: 10.1186/1471-2407-11-35 (PMC3039628; doi:10.1186/1471-2407-11-35)
Supplement: Additional file 3 — Characteristics of glioma studies selected for full text review [2,18,26,29-31,46,47,58,59,68,71,76,84,85,87,89,115,116,118,119,125-127]. [file 1471-2407-11-35-S3.DOC]

**Additional file 3:** **Characteristics of glioma studies selected for full text review**

| **Author / year** | **n / histology** | **Reference test**  **(% of informative results)** | **Percentage of methylated cases** | **Antibody for Index test** | **s.s association between test results** | **Cut-off value** | **Histological analysis of tissue^a^** | **Effect of methylated promoter/protein expression on survival** | | **Independent predictor on multivariate analysis** | **Included in the analysis** |
| --- | --- | --- | --- | --- | --- | --- | --- | --- | --- | --- | --- |
|  |  |  |  |  |  |  |  | PFS | OS |  |  |
| Karayan-Tapon et al, 2009 [46] | 81 GBM | MSP | 68% | Mouse monoclonal Ab clone MT 3.1 (Novus Biologicals) | yes | 15% | yes | no | no | no | no |
| Nakagawa et al, 2009 [115] | 11 GBM | MSP | 54.5% | Monoclonal Ab MAB16200 (Chemicon) | yes | >5% | yes | -- ^b^ | yes | yes | yes |
| Sonoda et al, 2009 [85] | 18 GBM | MSP | 25-79%^c^ | Mouse monoclonal Ab clone MT 3.1 (Chemicon) | yes | >10% | n.s. | yes | yes | yes | yes |
| Metellus et al, 2009 [84] | 21 GBM | MSP | 28.6% | Mouse monoclonal Ab clone MT 3.1 (Chemicon) | yes | >10% | n.s. | yes | yes | yes | yes |
| Cao et al, 2009 [116] | 73 GBM | MSP | 60.5% | Mouse monoclonal Ab clone MT 3.1 (Abcam, Camb.) | no | >5% | n.s. | yes | yes | yes | yes |
| Kuo et al, 2009 [118] | 36 LGG 13 AG | MSP | 53.1% | Mouse monoclonal Ab clone MT 3.1 (LabVision Corporation | no | >10% | n.s. | no | no | yes | yes |
| Felsberg et al, 2009 [119] | 57 GBM | MSP | 39% | Mouse monoclonal Ab clone MT 3.1 (Dako) | no | >10% | yes | yes | yes | yes | yes |
| Yang et al, 2009 [59] | 36 AG | MSP | 88.9% | Mouse monoclonal Ab clone MT 3.1 (NeoMarkers) | yes | >5% | n.s. | -- | -- | -- | no |
| Mellai et al, 2009 [58] | 96 GBM | MSP (97.1%) | 28.7% | Mouse monoclonal Ab clone MT 3.1 (Chemicon) | no | >50% | yes | -- | no | no | no |
| Preusser et al, 2008 [18] | 164 GBM | MSP | 48.8% | Mouse monoclonal Ab clone MT 3.1 (Dako)  Mouse monoclonal Ab clone MT23.2  (Zymed) | no (poor to moderate correlation) | neg vs. pos, 10%, 50% | n.s.^d^ | -- | yes | -- | no |
| Yachi et al, 2008 [30] | 25 GBM | MSP | 32% | Mouse monoclonal Ab clone MT 3.1 (Chemicon) | no | 5%, 10%, 20%, 30%, 35% | yes | no | no | -- | no |
| Sasai et al, 2008 [125] | 15 GBM  5 AG  1 LGG | MSP (85%) | 61% | Mouse monoclonal Ab clone MT 3.1 (Chemicon) | no | Qualitative | n.s. | -- | -- | -- | yes |
| Buccoliero at al, 2008 [126] | 12 AE | MSP (75%) | 0% | Mouse monoclonal Ab clone MT 3.1 (NeoMarkers) | yes | >10% | yes | -- | -- | -- | yes |
| Parkinson et al, 2008 [87] | 10 GBM | MSP (90.9%)  Sequencing | 30% | Mouse monoclonal Ab clone MT23.2 (Affinity Bioreagents) | yes | >10% | yes | -- | -- | -- | yes |
| Rodríguez et al, 2008 [29] | 50 GBM | MSP (78%) | 38.5% | Mouse monoclonal Ab clone MT 3.1 (NeoMarkers) | no | >10% | n.s. | -- | no | -- | yes |
| Jeuken et al, 2007 [26] | 33 gliomas | MSP | 21% | Mouse monoclonal Ab clone MT 3.1 (Chemicon) | no | Qualitative | n.s. | -- | no | -- | no |
| Grasbon-Frodl et al, 2007 [89] | 18 GBM  7 AA | MSP (100%)  Sequencing | 30-80% | Mouse monoclonal Ab clone MT 3.1 (Dako) | no | >10% | yes | -- | -- | -- | yes |
| Lavon et al, 2007 [76] | 10 O, 4 OA, 7 AO, 2 AOA | MSP | 19-68% | Monoclonal Ab MAB16200 (Chemicon) | no | >10% | n.s. | -- | -- | -- | yes |
| Cankovic et al, 2007 [127] | 80 GBM  43 AG  40 LGG | MSP (98%) | 44% | Mouse monoclonal Ab clone MT23.2 (Invitrogen) | yes | >5% | yes | -- | -- | -- | yes |
| Maxwell et al, 2006 [68] | 22 GBM  10 AG | Modified MSP  (96.8%) | 61% | Mouse monoclonal Ab clone MT 3.1 | no | >20% | yes | -- | -- | -- | yes |
| Brell et al, 2005 [31] | 40 AG | MSP (45%) | 50% | Mouse monoclonal Ab clone MT 3.1 (NeoMarkers) | no | >5% | yes | no | yes | yes | yes |
| Mölleman et al, 2005 [71] | 9 O  23 OA  9 AO  11 AOA | MSP (100%) | 88% | Mouse monoclonal Ab clone MT 3.1 (NeoMarkers) | no | >10% | yes | -- | -- | -- | yes |
| Rood et al, 2004 [47] | 32 Medulloblastoma | MSP (100%) | 76% | Mouse monoclonal Ab clone MT 3.1 (NeoMarkers) | no | Qualitative | n.s. | -- | -- | -- | no |
| Esteller et al. 1999 [2] | 8 gliomas | MSP | 38% | Mouse monoclocal AB (N99200, Novus Molecular Inc., San Diego) | yes | Qualitative | n.s. | -- | -- | -- | yes |

Abbreviations: s.s.: statistically significant; GBM: Glioblastoma Multiforme; AG: Anaplastic Glioma; LGG: Low Grade Glioma; AE: Anaplastic Ependymoma; AA: Anaplastic Astrocytoma; O: Oligodendroglioma; OA: Oligoastrocytoma; AO: Anaplastic Oligodendroglioma; AOA: Anaplastic Oligoastrocytoma; Ab: Antibody; a: Histological analysis of the tissue used for DNA extraction performed or not; b: not done; c: 79% for long term survivors and 25% for short term survivors; d: not stated. OS: Overall survival; PFS: Progression-free survival
