# Supplementary material for: O6-Methylguanine-DNA methyltransferase protein expression by immunohistochemistry in brain and non-brain systemic tumours: systematic review and meta-analysis of correlation with methylation-specific polymerase chain reaction
Source: BMC Cancer. 2011 Jan 26;11:35. doi: 10.1186/1471-2407-11-35 (PMC3039628; doi:10.1186/1471-2407-11-35)
Supplement: Additional file 4 — Additional file 1. Characteristics of non-glioma studies included in the analysis [1,32,63-67,69,70,72-75,77-83,91,95,96,128-139]. [file 1471-2407-11-35-S4.DOC]

**Additional file 4:** Characteristics of non-glioma studies included in the analysis

| **Author / year** | | **n / histology** | **Reference test (% of informative results)** | | **Percentage of methylated cases** | | | **Antibody for Index test** | **s.s. association between tests results** | | **Cut-off value** | **Histological analysis of tissue^a^** | **Effect of methylated promoter/protein expression on survival** | | **Independent predictor on multivariate analysis** | |
| --- | --- | --- | --- | --- | --- | --- | --- | --- | --- | --- | --- | --- | --- | --- | --- | --- |
|  |  |  |  |  |  |  |  |  |  |  |  |  | PFS | OS |  |  |
| Primary non-glial brain tumours | | | | | | | | | | | | | | | | |
| McCormack et al 2009 [65] | | 46 pituitary tumours | | MSP (72%) | 9% | Mouse monoclonal Ab clone MT23.2 (Affinity Bioreagents) | | | | yes | >10% | yes | -- | -- | | -- |
| Ingold et al. 2009 [32] | | 178 brain metastases | | Nested MSP (61.2%) | 29.6% | Mouse monoclonal Ab clone MT 3.1 (NeoMarkers) | | | | yes | >5% | n.s. | -- | -- | | -- |
| Chu et al. 2006 [69] | | 11 PCNSL | | Nested MSP | 52% | Mouse monoclonal Ab clone MT3.1 (Lab Vision Corporation) | | | | yes | >80% | yes | -- | -- | | -- |
| Non-brain systemic tumours | | | | | | | | | | | | | | | | |
| Kim et al. 2009 [128] | 62 soft tissue sarcomas | | MSP (100%) | | 33.9% | | Mouse monoclonal Ab clone MT 3.1 (NeoMarkers) | | | yes | >10% | n.s. | yes | yes | | yes |
| Uccella e al, 2009 [70] | 50 B lymphomas | | MSP | | 20% | | Mouse monoclonal Ab clone MT 3.1 (NeoMarkers) | | | yes | >5% | n.s. | yes | yes | | -- |
| Lee et al, 2009 [74] | 53 B lymphomas | | MSP | | 45.2% | | MT 5.1 ; BD Biosciences, Pharmingen, USA | | | yes | n.s. | n.s. | no | no | | no |
| Wu et al, 2009 [83] | 85 lung metastases and primary cancer | | Nested MSP | | 4-11% | | Mouse monoclonal Ab clone MT3.1 (Lab Vision) | | | yes | >10% | n.s. | yes | yes | | yes |
| Zou et al, 2009 [129] | 57 early gastric cancer | | MSP | | 48.1-50% | | Monoclonal Ab Zhongshan Biotech, China | | | yes | >5% | n.s. | -- | -- | | -- |
| Rimel et al. 2009 [64] | 141 endometrial and ovarian cancer | | COBRA | | 0% | | Mouse monoclonal Ab clone MT3.1 (Dako) | | | yes | Qualitative | n.s. | -- | -- | | -- |
| Kuester et al. 2009 [130] | 47 Barrett’s adeno-carcinoma | | MSP | | 78.9% | | Mouse monoclonal Ab clone MT 23.2 (Zymed) | | | yes | Immunore-active score | yes | -- | -- | | -- |
| Nagasaka et al. 2008 [66] | 85 colorrectal carcinoma, 29 adenomatous  polyps | | COBRA and sequencing | | 36% | | Mouse monoclonal Ab clone MT3.1 (PharMingen) | | | yes | Qualitative | n.s. | -- | -- | | -- |
| Herath et al. 2007 [75] | 36 hepato-cellular carcinoma | | MSP | | 0% | | Mouse monoclonal Ab clone MT3.1 (NeoMarkers) | | | no | Qualitative | n.s. | -- | -- | | -- |
| Mikami et al. 2007 [131] | 153 ulcerative colitis-associated tumours | | MSP | | 30-33% | | Mouse monoclonal Ab clone MT3.1 (NeoMarkers) | | | yes | Qualitative | yes | -- | -- | | -- |
| Ogawa et al. 2006 [78] | 34 colorectal carcinomas | | MSP | | 58.8% | | Mouse monoclonal Ab clone MT3.1 (NeoMarkers) | | | yes | >10% | yes | -- | -- | | -- |
| Baumann et al. 2006 [67] | 101 esophageal adeno-carcinoma | | RT-MSP | | 63.6% | | Mouse monoclonal Ab clone MT3.1 (Lab-Vision) | | | yes | >5% | yes | -- | no | | -- |
| Kawaguchi et al. 2006 [132] | 50 soft tissue sarcomas | | MSP | | 15% | | Mouse monoclonal Ab clone MT3.1 (Santa Cruz Biotechnology) | | | yes | >10% | yes | -- | -- | | -- |
| Martin et al. 2006 [77] | 20 monoclonal gammo-pathies | | MSP (100%) | | 23% | | Mouse monoclonal Ab clone MT3.1 (Dako) | | | yes | >30% | n.s. | -- | -- | | -- |
| Fox et al. 2006 [133] | 110 colorectal cancer | | MSP (100%) | | 43% | | Mouse monoclonal Ab clone MT3.1 (NeoMarkers) | | | yes | Qualitative | n.s | -- | -- | | -- |
| Munot et al. 2006 [72] | 18 breast cancer | | MSP | | 38.8% | | MGMT Pharmigen, Ox, UK | | | yes | Immunore-active score | yes | -- | -- | | -- |
| Shen et al. 2005 [134] | 24 colorectal carcinoma | | MSP | | 46% | | Monoclonal Ab MAB16200 (Chemicon) | | | yes | Qualitative | n.s. | -- | -- | | -- |
| Koga et al. 2005 [135] | 37 biliary tract cancers | | MSP (two-step method) | | 49% | | Rabbit polyclonal anti-MGMT antibody (clone G168-728; PharMingen, San Diego, CA) | | | yes | >10% | yes | -- | yes | | -- |
| Qi et al. 2005 [63] | 89 colorectal tumours | | MSP | | 40.7-43.5% | | Mouse anti-MGMT monoclonal Ab | | | yes | >10% | n.s. | -- | -- | | -- |
| Kohonen-Corish et al. 2005 [91] | 176 colon cancer | | MSP (98.9%) | | 53% | | Clone MT5.1, BD Pharmigen 557045 | | | yes | Qualitative | yes | -- | no | | -- |
| Rossi et al. 2004 [79] | 28 B lymphomas | | MSP | | 23.8-27.6% | | Mouse monoclonal Ab clone MT3.1 (Chemicon) | | | yes | Qualitative | n.s. | -- | -- | | -- |
| Kang et al. 2004 [136] | 14 PINs | | MSP | | 75.7% | | Mouse monoclonal Ab clone MT3.1 (Chemicon) Temecula, CA) | | | yes | Qualitative | n.s. | -- | -- | | -- |
| Zhang et al. 2003 [137] | 83 hepato-cellular carcinoma | | MSP | | 39% | | Mouse monoclonal Ab clone MT3.1 (NeoMarkers) | | | yes | Qualitative | n.s. | -- | -- | | -- |
| Kim et al. 2003 [80] | 169 colorectal adenomas and carcinoma | | MSP | | 51.4% | | Mouse monoclonal Ab clone MT3.1 (Chemicon) | | | yes | >10% | n.s | -- | -- | | -- |
| Bae et al. 2002 [1] | 149 gastric carcinoma | | MSP | | 14.1% | | Mouse monoclonal Ab clone MT3.1 (Chemicon) | | | yes | Qualitative | n.s. | -- | yes | | no |
| Choy et al. 2002 [81] | 23 retino-blastomas | | MSP | | 34.7% | | Ab. 3B8, Inst. Mol.Cell. Biology, Nat.Univ. of Singapore | | | yes | >10% | n.s. | -- | -- | | -- |
| Esteller et al. 2002 [138] | 26 B lymphoma | | MSP | | 36% | | Mouse monoclonal Ab clone MT3.1 (Chemicon) | | | yes | Qualitative | n.s. | yes | yes | | yes |
| Hayashi et al. 2002 [139] | 87 lung adeno-carcinoma | | MSP | | 35.6% | | Mouse monoclonal Ab clone MT3.1 (Chemicon) | | | yes | Immuno-reactive score | n.s. | -- | yes | | -- |
| Smith-Sørensen et al. 2002 [96] | 20 testicular carcinoma | | MSP | | 46% | | Goat polyclonal Ab sc-8825 (Santa Cruz Biotechnology) | | | no | >5% | yes | -- | -- | | -- |
| Park et al. 2001 [95] | 71 gastric carcinoma | | MSP | | 23% | | Mouse monoclonal Ab clone MT3.1 (NeoMarkers) | | | no | >5% | yes | yes | -- | | no |
| Wolf et al. 2001 [73] | 19 non-small cell lung cancer | | MSP | | 29% | | Mouse monoclonal Ab clone MT3.1 (NeoMarkers) | | | yes | Qualitative | yes | -- | -- | | -- |
| Whitehall et al. 2001 [82] | 80 colorectal cancer | | MSP | | 38.7% | | Mouse monoclonal Ab clone MT3.1 (NeoMarkers) | | | yes | Qualitative | n.s. | -- | -- | | -- |

Abbreviations: s.s.: statistically significant; COBRA: Combined Bisulfite Restriction Analysis; PINs: Prostatic Intraepithelial Neoplasia; Ab: Antibody; a: Histological analysis of the tissue used for DNA extraction performed or not; b: not done; c: 79% for long term survivors and 25% for short term survivors; d: not stated.
