# Supplementary material for: O6-Methylguanine-DNA methyltransferase protein expression by immunohistochemistry in brain and non-brain systemic tumours: systematic review and meta-analysis of correlation with methylation-specific polymerase chain reaction
Source: BMC Cancer. 2011 Jan 26;11:35. doi: 10.1186/1471-2407-11-35 (PMC3039628; doi:10.1186/1471-2407-11-35)
Supplement: Additional file 5 — Evaluation of quality of the included studies using the QUADAS tool [1,2,29,31,32,63-85,87,89,91,93,95,96,115,116,118,119,125-133,135-139]. [file 1471-2407-11-35-S5.DOC]

**Additional file 5: Evaluation of quality of the included studies using the QUADAS tool**

|  | Representative spectrum | Selection criteria | Reference standard | Partial verification bias | Differential verification bias | Execution of Reference S. | Execution of Index Test | Reference S. blinded | Index Test blinded | Uninterpretable Results | Withdrawal explained |
| --- | --- | --- | --- | --- | --- | --- | --- | --- | --- | --- | --- |
| Ucella et al, 2009 [70] | Yes | Yes | Yes | Uncl. | Yes | Yes | Uncl. | no | No | Uncl | Uncl. |
| Wu et al, 2009 [83] | Yes | Yes | Yes | No | Yes | Yes | Yes | No | No | Uncl | Uncl |
| Zou et al, 2009 [129] | Yes | Yes | Yes | Yes | Yes | Yes | Yes | Yes | Yes | Yes | Yes |
| Sonoda et al, 2009 [85] | Yes | Yes | Yes | No | Yes | Yes | Yes | Yes | Yes | Uncl. | Uncl. |
| Lee et al, 2009 [74] | Yes | Yes | Yes | Yes | Yes | Yes | No | No | No | Uncl. | Uncl. |
| Metellus et al, 2009 [84] | Yes | Yes | Yes | Uncl. | Yes | Yes | Yes | No | No | Uncl. | Uncl. |
| Cao et al, 2009 [116] | Yes | Uncl. | Yes | Uncl. | Yes | Yes | Yes | No | No | Yes | Yes |
| Kuo et al, 2009 [118] | Yes | Yes | Yes | Yes | Yes | Yes | Uncl | No | No | Yes | Yes |
| Felsberg et al, 2009 [119] | Yes | Yes | Yes | Yes | Yes | Yes | Yes | Uncl. | Uncl. | Yes | Yes |
| Ingold et al, 2009 [32] | Yes | Yes | Uncl. | No | Yes | Yes | Yes | Uncl. | Uncl. | Yes | Yes |
| Kim et al, 2009 [128] | Yes | Yes | Yes | Yes | Yes | Yes | Yes | Uncl. | Uncl. | Yes | Yes |
| Rimel et al, 2009 [64] | Yes | Uncl. | Uncl. | Uncl. | Yes | Yes | Uncl. | Uncl. | Uncl. | Uncl. | Yes |
| Nakagawa et al, 2009 [115] | Yes | Yes | Yes | Uncl. | Yes | Yes | Yes | Uncl. | Yes | Yes | Yes |
| Buccoliero et al, 2008 [126] | Yes | Yes | Yes | Yes | Yes | Yes | Yes | Uncl. | Uncl. | Yes | Yes |
| Kuester et al, 2008 [130] | Yes | Yes | Yes | Yes | Yes | Yes | Yes | Uncl. | Uncl. | Yes | Yes |
| McCormack et al, 2008 [65] | Yes | Yes | Uncl. | No | Yes | Yes | Yes | No | Yes | Yes | Yes |
| Nagasaka et al, 2008 [66] | Yes | Yes | Uncl. | No | Yes | Yes | Yes | Uncl | Uncl. | Uncl. | Yes |
| Parkinson et al, 2008 [87] | Yes | Yes | Yes | No | Yes | Yes | Yes | Yes | Yes | Yes | Yes |
| Rodriguez et al, 2008 [29] | Yes | Uncl. | Yes | Yes | Yes | Yes | Yes | Uncl. | Yes | Yes | Yes |
| Sasai et al, 2008 [125] | Yes | Yes | Yes | No | Yes | Yes | Yes | Uncl. | Uncl. | Yes | Yes |
| Cankovic et al, 2007 [127] | Yes | Yes | Yes | Yes | Yes | Yes | Yes | Yes | Yes | Yes | Yes |
| Grasbon-Frodl et al, 2007 [89] | Yes | Yes | Yes | Yes | Yes | Yes | Yes | Uncl. | Yes | Yes | Yes |
| Herath et al, 2007 [75] | Yes | Uncl. | Yes | Yes | Yes | Yes | Yes | Uncl. | Uncl. | Uncl. | Yes |
| Lavon et al, 2007 [76] | Yes | Uncl. | Yes | No | Yes | Yes | Yes | Uncl. | Yes | Uncl. | Yes |
| Mikami et al, 2007 [131] | Yes | Uncl. | Yes | Yes | Yes | Yes | Yes | Uncl. | Uncl. | Yes | Yes |
| Baumann et al, 2006 [67] | Yes | Yes | Uncl. | Yes | Yes | Yes | Yes | Yes | Yes | Yes | Yes |
| Chu et al, 2006 [69] | Yes | Yes | Uncl. | Yes | Yes | Yes | Uncl. | Uncl. | Uncl. | Yes. | Yes |
| Fox et al, 2006 [133] | Yes | Yes | Yes | Yes | Yes | Yes | Yes | Uncl. | Uncl. | Yes | Yes |
| Kawaguchi et al, 2006 [132] | Yes | Yes | Yes | Yes | Yes | Yes | Yes | Uncl. | Uncl. | Yes | Yes |
| Martin et al, 2006 [77] | Yes | Uncl. | Yes | Yes | Yes | Yes | Yes | Uncl. | Uncl. | Uncl. | Yes |
| Maxwell et al, 2006 [68] | Yes | Yes | Uncl. | No | Yes | Yes | Yes | Uncl. | Uncl. | Yes | Yes |
| Munot et al, 2006 [72] | Yes | Yes | Yes | No | Yes | Yes | Uncl. | No | Yes | Yes | Yes |
| Ogawa et al, 2006 [78] | Yes | Yes | Yes | Uncl. | Yes | Yes | Yes | Uncl. | Uncl. | Uncl. | Yes |
| Brell et al, 2005 [31] | Yes | Yes | Yes | No | Yes | Yes | Yes | Yes | Yes | Yes | Yes |
| Kohonen et al, 2005 [91] | Yes | Yes | Yes | Yes | Yes | Yes | Yes | Uncl. | Uncl. | Yes | Yes |
| Koga et al, 2005 [135] | Yes | Yes | Yes | Yes | Yes | Yes | Yes | Uncl. | Uncl. | Yes | Yes |
| Mölleman et al, 2005 [71] | Yes | Yes | Yes | No | Yes | Yes | Yes | Uncl. | Uncl. | Yes | Yes |
| Shen et al, 2005 [93] | Yes | Yes | Yes | Yes | Yes | Yes | Yes | Uncl. | Uncl. | Yes | Yes |
| Qi et al, 2005 [63] | Yes | Yes | Yes | Yes | Yes | Yes | Yes | Uncl. | Uncl. | Yes | Yes |
| Kang et al, 2004 [118] | Yes | Yes | Yes | Yes | Yes | Yes | Yes | Uncl. | Uncl. | Yes | Yes |
| Rossi et al, 2004 [79] | Yes | Yes | Yes | Yes | Yes | Yes | Yes | Yes | Uncl. | Uncl. | Yes |
| Kim et al, 2003 [80] | Yes | Yes | Yes | No | Yes | Yes | Yes | No | Uncl. | Uncl. | Yes |
| Zhang et al, 2003 [137] | Yes | Yes | Uncl. | Yes | Yes | Yes | Yes | Yes | No | Yes | Yes |
| Bae et al, 2002 [1] | Yes | Yes | Yes | Yes | Yes | Yes | Uncl. | Uncl. | Uncl. | Yes | Yes |
| Choy et al, 2002 [81] | Yes | Uncl. | Yes | Yes | Yes | Yes | Yes | Uncl. | Uncl. | Uncl. | Yes |
| Esteller et al, 2002 [138] | Yes | Yes | Yes | Yes | Yes | Yes | Yes | Uncl. | Yes | Yes | Yes |
| Hayashi et al, 2002 [139] | Yes | Yes | Yes | Yes | Yes | Yes | Yes | Uncl. | Uncl. | Yes | Yes |
| Smith-Sorensen et al, 2002 [96] | Yes | Uncl. | Yes | Yes | Yes | Yes | Yes | Yes | No | Yes | Yes |
| Park et al, 2001 [95] | Yes | Yes | Yes | Yes | Yes | Yes | Yes | Uncl. | Uncl. | Yes | Yes |
| Whitehall et al, 2001 [82] | Yes | Yes | Yes | Yes | Yes | Yes | Yes | Uncl. | Uncl. | No | Yes |
| Wolf et al, 2001 [73] | Yes | Yes | Yes | Uncl. | Yes | Yes | Uncl. | Uncl. | Uncl. | Uncl. | Yes |
| Esteller et al, 1999 [2] | Yes | Uncl. | Yes | Uncl. | Yes | Yes | Yes | Uncl. | Yes | Uncl. | Yes |
