# Supplementary material for: O6-Methylguanine-DNA methyltransferase protein expression by immunohistochemistry in brain and non-brain systemic tumours: systematic review and meta-analysis of correlation with methylation-specific polymerase chain reaction
Source: BMC Cancer. 2011 Jan 26;11:35. doi: 10.1186/1471-2407-11-35 (PMC3039628; doi:10.1186/1471-2407-11-35)
Supplement: Additional file 6 — Tabular results of Sensitivity, Specificity, Likelihood Ratios, and Diagnostic Odds Ratio [1,2,29,31,32,63-87,89,91,95,96,115,116,118,119,125-139]. [file 1471-2407-11-35-S6.DOC]

**Additional file 6: Tabular results of Sensitivity, Specificity, and Likelihood Ratios, and Diagnostic Odds Ratio**

Study TP FP FN TN Sens. [95% CI] Spec. [95% CI] LR- [95% CI] LR+ [95% CI] DOR [95% CI]

--------------------------------------------------------------------------------------------­­­­------------------------------------------------------------------------------------------------------------------------------------

Felsberg 2009 [119] 19 21 5 12 0.722 0.578 - 0.929 0.634 0.204 - 0.549 0.576 0.233 - 1.411 1.244 0.895 - 1.730 2.171 0.645 - 7.310

Kuo 2009 [118] 11 9 15 14 0.423 0.234 - 0.631 0.609 0.285 - 0.803 0.948 0.596 - 1.508 1.081 0.548 - 2.132 1.141 0.364 - 3.578

Cao 2009 [116] 31 13 13 16 0.705 0.548 - 0.832 0.552 0.357 - 0.736 0.536 0.305 - 0.939 1.572 1.005 - 2.457 2.935 1.105 - 7.798

Metellus 2009 [84] 6 2 0 13 1.000 0.541 - 1.000 0.687 0.595 - 0.983 0.085 0.006 - 1.234 5.943 1.869 - 18.901 70.200 2.927 - 1683.5

Sonoda 2009 [85] 8 1 2 7 0.800 0.444 - 0.975 0.875 0.473 - 0.997 0.229 0.064 - 0.811 6.400 0.997 - 41.087 28.000 2.067 - 379.25

Nakagawa 2009 [115] 5 0 1 5 0.833 0.359 - 0.996 1.000 0.478 - 1.000 0.234 0.055 - 0.986 9.429 0.645 - 137.77 40.333 1.330 - 1223.0

Sasai 2008 [125] 5 2 6 5 0.455 0.167 - 0.766 0.714 0.290 - 0.963 0.764 0.374 - 1.560 1.591 0.417 - 6.065 2.083 0.275 - 15.772

Buccoliero 2008 [126] 0 0 0 12 0.500 0.000 - 1.000 0.962 0.693 - 1.000 0.520 0.073 - 3.703 13.00 0.456 - 370.89 25.000 0.199 - 3139.1

Parkinson 2008 [87] 2 1 1 4 0.667 0.094 - 0.992 0.800 0.284 - 0.995 0.417 0.079 - 2.190 3.333 0.485 - 22.897 8.000 0.310 - 206.37

McCormack 2009 [65] 3 8 1 34 0.750 0.194 - 0.994 0.810 0.659 - 0.914 0.309 0.056 - 1.697 3.938 1.697 - 9.138 12.750 1.168 - 139.24

Rodriguez 2008 [29] 14 18 1 6 0.933 0.681 - 0.998 0.250 0.098 - 0.467 0.267 0.036 - 2.003 1.244 0.952 - 1.626 4.667 0.502 - 43.367

Grasbon-Frodl 2007 [89] 4 4 6 9 0.400 0.122 - 0.738 0.692 0.386 - 0.909 0.867 0.465 - 1.615 1.300 0.427 - 3.961 1.500 0.266 - 8.449

Lavon 2007 [76] 3 4 0 5 1.000 0.292 - 1.000 0.556 0.212 - 0.863 0.227 0.016 - 3.226 1.944 0.892 - 4.237 8.556 0.344 - 212.94

Cankovic 2007 [127] 8 2 3 9 0.727 0.390 - 0.940 0.818 0.482 - 0.977 0.333 0.122 - 0.910 4.000 1.085 - 14.748 12.000 1.581 - 91.085

Maxwell 2006 [68] 7 1 12 8 0.368 0.163 - 0.616 0.889 0.518 - 0.997 0.711 0.470 - 1.075 3.316 0.477 - 23.060 4.667 0.478 - 45.546

Brell 2005 [31] 7 4 13 16 0.350 0.154 - 0.592 0.800 0.563 - 0.943 0.813 0.551 - 1.199 1.750 0.606 - 5.054 2.154 0.515 - 9.000

Möllemann 2005 [71] 19 3 18 3 0.514 0.344 - 0.681 0.500 0.118 - 0.882 0.973 0.409 - 2.313 1.027 0.435 - 2.426 1.056 0.188 - 5.926

Ingold 2009 [32] 26 35 22 95 0.542 0.392 - 0.686 0.731 0.646 - 0.805 0.627 0.453 - 0.868 2.012 1.370 - 2.956 3.208 1.613 - 6.379

Chu 2006 [69] 7 1 0 3 1.000 0.590 - 1.000 0.750 0.194 - 0.994 0.089 0.006 - 1.389 3.125 0.809 - 12.064 35.000 1.119 - 1094.7

Kuester 2008 [130] 7 0 30 10 0.189 0.080 - 0.352 1.000 0.692 - 1.000 0.841 0.686 - 1.031 4.342 0.269 - 70.198 5.164 0.271 - 98.402

Nagasaka 2008 [66] 16 8 8 82 0.667 0.447 - 0.844 0.911 0.832 - 0.961 0.366 0.207 - 0.647 7.500 3.653 - 15.399 20.500 6.710 - 62.631

Herath 2007 [75] 0 4 0 32 0.500 0.000 - 1.000 0.878 0.729 - 0.962 0.569 0.080 - 4.056 4.111 0.482 - 35.038 7.222 0.127 - 411.24

Baumann 2006 [67] 33 4 19 45 0.635 0.490 - 0.764 0.918 0.804 - 0.977 0.398 0.275 - 0.575 7.774 2.972 - 20.334 19.539 6.076 - 62.833

Kawaguchi 2006 [132] 7 6 3 34 0.700 0.348 - 0.933 0.850 0.702 - 0.943 0.353 0.136 - 0.918 4.667 2.011 - 10.831 13.222 2.651 - 65.951

Shen 2005 [134] 8 0 6 10 0.571 0.289 - 0.823 1.000 0.692 - 1.000 0.454 0.251 - 0.821 12.46 0.802 - 193.85 27.462 1.347 - 560.06

Rossi 2004 [79] 17 0 0 11 1.000 0.805 - 1.000 1.000 0.715 - 1.000 0.029 0.002 - 0.447 23.33 1.545 - 352.29 805.00 14.893 - 43510.8

Kang 2004 [136] 13 0 0 1 1.000 0.753 - 1.000 1.000 0.025 - 1.000 0.048 0.003 - 0.813 3.857 0.349 - 42.628 81.000 1.135 - 5778.7

Kim 2003 [80] 72 4 15 78 0.828 0.732 - 0.900 0.951 0.880 - 0.987 0.181 0.114 - 0.288 16.96 6.492 - 44.335 93.600 29.681 - 295.17

Bae 2002 [1] 13 4 8 124 0.619 0.384 - 0.819 0.969 0.922 - 0.991 0.393 0.228 - 0.679 19.81 7.134 - 55.004 50.375 13.332 - 190.34

Esteller 2002 [138] 17 0 0 9 1.000 0.805 - 1.000 1.000 0.664 - 1.000 0.029 0.002 - 0.451 19.44 1.303 - 290.13 665.00 12.195 - 36261.5

Hayashi 2002 [139] 29 0 2 56 0.935 0.786 - 0.992 1.000 0.936 - 1.000 0.079 0.024 - 0.259 105.0 6.642 - 1662.9 1333.4 61.970 - 28690.6

Smith-Sorensen 2002 [96]6 7 1 6 0.857 0.421 - 0.996 0.462 0.192 - 0.749 0.310 0.046 - 2.084 1.592 0.885 - 2.863 5.143 0.475 - 55.642

Park 2001 [95] 5 9 13 44 0.278 0.097 - 0.535 0.830 0.702 - 0.919 0.870 0.637 - 1.188 1.636 0.630 - 4.245 1.880 0.535 - 6.604

Esteller 1999 [2] 3 0 0 5 1.000 0.292 - 1.000 1.000 0.478 - 1.000 0.136 0.010 - 1.843 10.50 0.720 - 153.07 77.000 1.223 - 4848.9

Choy 2002 [81] 6 1 2 14 0.750 0.349 - 0.968 0.933 0.681 - 0.998 0.268 0.080 - 0.896 11.25 1.624 - 77.920 42.000 3.170 - 556.48

Rimel 2009 [64] 0 0 1 140 0.250 0.000 - 0.939 0.996 0.967 - 1.000 0.753 0.338 - 1.675 70.50 1.809 - 2748.1 93.667 1.353 - 6483.3

Kim 2009 [128] 14 6 7 35 0.667 0.430 - 0.854 0.854 0.708 - 0.944 0.390 0.210 - 0.724 4.556 2.049 - 10.126 11.667 3.328 - 40.895

Koga 2005 [135] 16 4 2 15 0.889 0.653 - 0.986 0.789 0.544 - 0.939 0.141 0.037 - 0.531 4.222 1.741 - 10.240 30.000 4.775 - 188.47

Mikami 2007 [131] 14 1 17 121 0.452 0.273 - 0.640 0.992 0.955 - 1.000 0.553 0.402 - 0.761 55.09 7.531 - 403.10 99.647 12.309 - 806.70

Martin 2006 [77] 5 7 0 18 1.000 0.478 - 1.000 0.720 0.506 - 0.879 0.117 0.008 - 1.683 3.178 1.659 - 6.088 27.133 1.328 - 554.24

Kohonen-Corish 2005 [91]43 5 51 77 0.457 0.354 - 0.563 0.939 0.863 - 0.980 0.578 0.476 - 0.701 7.502 3.120 - 18.041 12.984 4.818 - 34.992

Whitehall 2001 [82] 23 10 8 39 0.742 0.554 - 0.881 0.796 0.657 - 0.898 0.324 0.176 - 0.599 3.635 2.014 - 6.563 11.213 3.873 - 32.459

Zhang 2003 [137] 30 3 2 48 0.938 0.792 - 0.992 0.941 0.838 - 0.988 0.066 0.017 - 0.255 15.93 5.297 - 47.948 240.00 37.872 - 1520.9

Wolf 2001 [73] 8 0 2 9 0.800 0.444 - 0.975 1.000 0.664 - 1.000 0.239 0.080 - 0.718 15.45 1.017 - 234.74 64.600 2.702 - 1544.5

Qi 2005 [63] 29 5 9 46 0.763 0.598 - 0.886 0.902 0.786 - 0.967 0.263 0.147 - 0.468 7.784 3.323 - 18.232 29.644 9.037 - 97.239

Fox 2006 [133] 27 10 20 53 0.574 0.422 - 0.717 0.841 0.727 - 0.921 0.506 0.357 - 0.717 3.619 1.948 - 6.724 7.155 2.940 - 17.412

Ogawa 2006 [78] 16 6 4 8 0.800 0.563 - 0.943 0.571 0.289 - 0.823 0.350 0.130 - 0.939 1.867 0.981 - 3.552 5.333 1.162 - 24.469

Munot 2006 [72] 7 0 0 7 1.000 0.590 - 1.000 1.000 0.590 - 1.000 0.067 0.005 - 0.982 15.00 1.018 - 220.92 225.00 3.926 - 12895.0

Uccella 2009 [70] 8 11 2 29 0.800 0.444 - 0.975 0.725 0.561 - 0.854 0.276 0.079 - 0.967 2.909 1.611 - 5.253 10.545 1.931 - 57.600

Wu 2009 [83] 4 0 0 81 1.000 0.398 - 1.000 1.000 0.955 - 1.000 0.101 0.007 - 1.395 147.60 9.168 - 2376.2 1467.0 25.992 - 82799.5

Zou 2009 [129] 14 6 8 29 0.636 0.407 - 0.828 0.829 0.664 - 0.934 0.439 0.247 - 0.778 3.712 1.678 - 8.211 8.458 2.458 - 29.107

Lee 2009 [74] 16 4 8 25 0.667 0.447 - 0.844 0.862 0.683 - 0.961 0.387 0.216 - 0.694 4.833 1.864 - 12.533 12.500 3.226 - 48.429

---------------------------------------------------------------------------------------------------------------------------------------------------------------------------------------------------------------------------------------------

Pooled Sens. 0.660[0.631-0.688] Pooled Spec. 0.851[0.834-0.868] Pooled LR- 0.404[0.331-0.493] Pooled LR+ 4.051[3.0-5.47] Pooled DOR 12.9[8.433-19.975]

------------------------------------------------------------------------------------------------------------------------------------------------------------------------------------------------------------------------------------------------

Abbreviations: TP: True Positive; FP: False Positive; FN: False Negative; TN: True Negative; Sens.: Sensitivity; Spec.: Specificity; CI: Confidence Interval; LR+: Positive Likelihood Ratio; LR+: Negative Likelihood Ratio; DOR: Diagnostic Odds Ratio
