# Supplementary material for: O6-Methylguanine-DNA methyltransferase protein expression by immunohistochemistry in brain and non-brain systemic tumours: systematic review and meta-analysis of correlation with methylation-specific polymerase chain reaction
Source: BMC Cancer. 2011 Jan 26;11:35. doi: 10.1186/1471-2407-11-35 (PMC3039628; doi:10.1186/1471-2407-11-35)
Supplement: Additional file 7 — Results of meta-regression analysis for all studies. [file 1471-2407-11-35-S7.DOC]

**Additional file 7: Results of meta-regression analysis for all studies.**

**Meta-Regression (Inverse Variance weights) (1)**

Covariate Coeff. Std. Err. p RDOR [95%CI]

-----------------------------------------------------------------------------------------------------

Cte. 1.650 0.4579 0.0008 ---- ----

S 0.012 0.1262 0.9273 ---- ----

Brain tumour *vs* others 1.702 0.4294 0.0003 5.49 [2.31-13.03]

Type of antibody -0.110 0.2486 0.6599 0.90 [0.54-1.46]

Paraffin *vs* frozen tissue -0.128 0.2657 0.6315 0.88 [0.51-1.50]

**Meta-Regression (Inverse Variance weights) (2)**

Covariate Coeff. Std. Err. p RDOR [95%CI]

-----------------------------------------------------------------------------------------------------

Cte. 1.521 0.3667 0.0001 ---- ----

S 0.010 0.1243 0.9393 ---- ----

Brain tumour *vs* others 1.648 0.4030 0.0002 5.19 [2.31-1.70]

Paraffin *vs* frozen tissue -0.135 0.2600 0.6057 0.87 [0.52-1.47]

**Meta-Regression (Inverse Variance weights)**

Covariate Coeff. Std. Err. p RDOR [95%CI]

-----------------------------------------------------------------------------------------------------

Cte. 1.406 0.2976 0.0000 ---- ----

S 0.001 0.1222 0.9939 ---- ----

Brain tumour *vs* others 1.679 0.3947 0.0001 5.36 [2.42-11.86]

-----------------------------------------------------------------------------------------------------

Abbreviations: RDOR: Relative Diagnostic Odds Ratio of the corresponding covariate; Coeff: Coefficient; Std. Err: Standard Error; S: S coefficient; Coeff: Covariate coefficient; Cte: Constant coefficient.
