# Supplementary material for: O6-Methylguanine-DNA methyltransferase protein expression by immunohistochemistry in brain and non-brain systemic tumours: systematic review and meta-analysis of correlation with methylation-specific polymerase chain reaction
Source: BMC Cancer. 2011 Jan 26;11:35. doi: 10.1186/1471-2407-11-35 (PMC3039628; doi:10.1186/1471-2407-11-35)
Supplement: Additional 8 — Results of meta-regression analysis for the subgroup of studies using semiquantitative scoring for IHC assessment. [file 1471-2407-11-35-S8.DOC]

**Additional file 8: Results of meta-regression analysis for the subgroup of studies using semiquantitative scoring for IHC assessment.**

**Meta-Regression (Inverse Variance weights) (1)**

Covariate Coeff. Std. Err. p RDOR [95%CI]

-----------------------------------------------------------------------------------------------------

Cte. 0.191 0.5048 0.0252 ---- ----

S -0.012 0.1520 0.9372 ---- ----

Brain tumour *vs* others 1.299 0.5068 0.0158 3.67 [1.30-10.34]

Type of antibody 0.025 0.3022 0.9358 1.02 [0.55-1.90]

Paraffin *vs* frozen tissue -0.054 0.2893 0.8541 0.95 [0.52-1.71]

Cut-off 0.329 0.2146 0.1362 1.39 [0.90-2.15]

**Meta-Regression (Inverse Variance weights) (2)**

Covariate Coeff. Std. Err. p RDOR [95%CI]

-----------------------------------------------------------------------------------------------------

Cte. 1.348 0.4925 0.0103 ---- ----

S -0.010 0.1509 0.9417 ---- ----

Brain tumour *vs* others 1.341 0.5047 0.0125 3.82 [1.36-10.71]

Type of antibody 0.127 0.2939 0.6677 1.14 [0.62-2.07]

Paraffin *vs* frozen tissue -0.114 0.2857 0.6939 0.89 [0.50-1.60]

**Meta-Regression (Inverse Variance weights) (3)**

Covariate Coeff. Std. Err. p RDOR [95%CI]

-----------------------------------------------------------------------------------------------------

Cte. 1.259 0.4445 0.0081 ---- ----

S -0.009 0.1487 0.9506 ---- ----

Brain tumour *vs* others 1.395 0.4802 0.0067 4.03 [1.52-10.77]

Type of antibody 0.111 0.2848 0.6996 1.12 [0.63-2.00]

**Meta-Regression (Inverse Variance weights) (4)**

Covariate Coeff. Std. Err. p RDOR [95%CI]

-----------------------------------------------------------------------------------------------------

Cte. 1.376 0.3022 0.0001 ---- ----

S -0.003 0.1466 0.9811 ---- ----

Brain tumour *vs* others 1.478 0.4306 0.0017 4.38 [1.82-10.54]

----------------------------------------------------------------------

Abbreviations: RDOR: Relative Diagnostic Odds Ratio of the corresponding covariate; Coeff: Coefficient; Std. Err: Standard Error; S: S coefficient; Coeff: Covariate coefficient; Cte: Constant coefficient.
