# Supplementary material for: O6-Methylguanine-DNA methyltransferase protein expression by immunohistochemistry in brain and non-brain systemic tumours: systematic review and meta-analysis of correlation with methylation-specific polymerase chain reaction
Source: BMC Cancer. 2011 Jan 26;11:35. doi: 10.1186/1471-2407-11-35 (PMC3039628; doi:10.1186/1471-2407-11-35)
Supplement: Additional file 9 — Summary of results. [file 1471-2407-11-35-S9.DOC]

**Additional file 9: Summary of results**

| **Subgroup**  **(nº of studies)** | | **Spearman correlation coefficient** | **Pooled Sensitivity**  **[CI]** | **Pooled Specificity**  **[CI]** | **Pooled LR+**  **[CI]** | **Pooled LR-**  **[CI]** | **Pooled DOR**  **[CI]** |
| --- | --- | --- | --- | --- | --- | --- | --- |
| **Semiquantitative IHC scoring** (36) | | 0.037  p = 0.833 | 0.666  [0.630-0.700] | 0.802  [0.777-0.826] | 3.156  [2.315-4.301] | 0.10  [0.315-0.534] | 9.494  [5.755-15.664] |
| Heterogeneity test | | | chi squared = 150.58  p < 0,001 | chi-squared = 202.01  p < 0,001 | Cochran-Q = 169.80  p < 0,001 | Cochran-Q = 160.93  p < 0,001 | Cochran-Q = 99.09  p < 0,001 |
| Inconsistency test | | | I-square = 77.4 % | I-square = 83.2 % | I-square = 80 % | I-square = 78.9 % | I-square = 65.7 % |
| **Brain tumours + semiquantitative IHC scoring** (14) | | 0.113  p = 0.666 | 0.614  [0.556–0.670] | 0.671  [0.622–0.718] | 1.800  [1.400–2.320] | 0.630  [0.500–0.780] | 3.470  [2.180–5.530] |
| Heterogeneity test | | | chi squared = 51.51  p < 0.001 | chi squared = 54.99  p < 0.001 | Cochran-Q = 30.22  p = 0.016 | Cochran-Q = 22.33  p = 0.132 | Cochran-Q = 30.34  p = 0.205 |
| Inconsistency test | | | I-square = 68.9 % | I-square = 70.9 % | I-square = 47.1 % | I-square = 28.4 % | I-square = 21.3 % |
|  | ***Cut-off*  > 5%** (4) | | 0.597  [0.507–0.682] | 0.723  [0.655–0.785] | 1.900  [1.450–2.500] | 0.600  [0.450–0.810] | 3.420  [2.060–5.670] |
| Heterogeneity test | | | chi squared = 10.14  p = 0.038 | chi squared = 8.32  p = 0.800 | Cochran-Q = 3.58  p = 0.465 | Cochran-Q = 5.70  p = 0.223 | Cochran-Q = 4.02  p = 0.402 |
| Inconsistency test | | | I-square = 60.6 % | I-square = 51.9 % | I-square = 0.0 % | I-square = 29.8 % | I-square = 0.6 % |
|  | ***Cut-off*  > 10%** (9) | | 0.645  [0.559–0.724] | 0.601  [0.525–0.674] | 1.70  [1.200–2.330] | 0.600  [0.390–0.930] | 3.390  [1.540–7.430] |
| Heterogeneity test | | | chi squared = 29.16  p = 0.001 | chi squared = 37.94  p < 0.001 | Cochran-Q = 19.43  p = 0.021 | Cochran-Q = 14.06  p = 0.120 | Cochran-Q = 13.94  p = 0.124 |
| Inconsistency test | | | I-square = 69.1 % | I-square = 76.3 % | I-square = 53.7 % | I-square = 36 % | I-square = 35.4% |
|  | ***Cut-off*  > 20%** (1) | | 0.538  [0.334– 0.734] | 0.846  [0.546–0.981] | 3.186  [1.052–9.653] | 0.333  [0.027–4.152] | 8.620  [1.289–57.628] |
| Heterogeneity test | | | chi squared = 10.88  p = 0.001 | chi squared = 0.39  p = 0.535 | Cochran-Q = 0.00  p = 0.960 | Cochran-Q = 3.57  p = 0.059 | Cochran-Q = 0.92  p = 0.338 |
| Inconsistency test | | | I-square = 90.8 % | I-square = 0.0 % | I-square = 0.0 % | I-square = 71.9 % | I-square = 0.0% |
| **Other tumours + semiquantitative IHC scoring** (18) | | -0.029  p = 0.909 | 0.702  [0.655–0.745] | 0.79  [0.851–0.903] | 4.790  [3.070–7.470] | 0.310  [0.200–0.490] | 18.240  [9.550–34.830] |
| Heterogeneity test | | | chi squared = 93.19  p < 0.001 | chi squared = 82.87  p < 0.001 | Cochran-Q = 64.12  p < 0.001 | Cochran-Q = 147.89  p < 0.001 | Cochran-Q = 42.37  p < 0.001 |
| Inconsistency test | | | I-square = 81.8% | I-square = 79.5 % | I-square = 73.5 % | I-square = 88.5 % | I-square = 59.9 % |
|  | ***Cut-off*  > 5%** (3) | | 0.606  [0.507–0.698] | 0.805  [0.742–0.859] | 2.830  [1.590–5.060] | 0.490  [0.290–0.820] | 7.220  [2.950–17.690] |
| Heterogeneity test | | | chi squared = 12.08  p = 0.017 | chi squared = 14.28  p = 0.006 | Cochran-Q = 11.93  p = 0.017 | Cochran-Q = 15.56  p = 0.003 | Cochran-Q = 7.55  p = 0.109 |
| Inconsistency test | | | I-square = 66.9 % | I-square = 72 % | I-square = 66.5 % | I-square = 74.3 % | I-square = 47 % |
|  | ***Cut-off*  > 10%** (8) | | 0.796  [0.735–0.849] | 0.907  [0.871–0.935] | 6.350  [3.210–12.550] | 0.250  [0.190–0.330] | 26.210  [11.210–61.260] |
| Heterogeneity test | | | chi squared = 6.25  p = 0.511 | chi squared = 34.06  p < 0.001 | Cochran-Q = 26.66  p < 0.001 | Cochran-Q = 6.17  p = 0.520 | Cochran-Q = 15.26  p = 0.032 |
| Inconsistency test | | | I-square = 0.0 % | I-square = 79.4% | I-square = 73.7 % | I-square = 0.0 % | I-square = 54.1 % |
|  | ***Cut-off*  > 20%★** (1) | | 1.000 [0.478–1.000] | 0.720 [0.506–0.879] | 3.178  [1.659–6.088] | 0.117  [0.008–1.683] | 27.133  [1.328–554.24] |
| **Qualitative IHC scoring** (14) | 0.008  p = 0.979 | | 0.649  [0.598–0.698] | 0.932  [0.910–0.951] | 7.620  [4.670–12.440] | 0.390  [0.239–0.520] | 27.760  [13.230–58.270] |
| Heterogeneity test | | | chi squared = 83.40  p < 0.001 | chi squared = 40.08  p < 0.001 | Cochran-Q = 27.10  p = 0.012 | Cochran-Q = 40.88  p < 0.001 | Cochran-Q = 30.420  p = 0.004 |
| Inconsistency test | | | I-square = 84.4 % | I-square = 67.6 % | I-square = 52.5 % | I-square = 68.2 % | I-square = 57.3 % |
| **Brain tumours + qualitative IHC scoring** (2) | | n.a. | 0.571  [0.289–0.823] | 0.833  [0.516–0.979] | 2.891  [0.486–17.176] | 0.441  [0.063–3.065] | 7.942  [0.256–245.97] |
| Heterogeneity test | | | chi squared = 3.96  p = 0.047 | chi squared = 2.44  p = 0.118 | Cochran-Q = 1.64  p = 0.201 | Cochran-Q = 2.37  p = 0.123 | Cochran-Q = 2.38  p = 0.123 |
| Inconsistency test | | | I-square = 74.8 % | I-square = 59 % | I-square = 38.9 % | I-square = 57.9 % | I-square = 57.9 % |
| **Other tumours + qualitative IHC scoring** (12) | | 0.195  p = 0.544 | 0.652  [0.600–0.720] | 0.934  [0.912–0.953] | 8.500  [5.150–14.040] | 0.370  [0.270–0.510] | 32.23  [15.190–68.370] |
| Heterogeneity test | | | chi squared = 79.06  p < 0.001 | chi squared = 36.24  p < 0.001 | Cochran-Q = 22.67  p = 0.019 | Cochran-Q = 37.73  p < 0.001 | Cochran-Q = 25.34  p = 0.008 |
| Inconsistency test | | | I-square = 86.1 % | I-square = 69.6 % | I-square = 51.5 % | I-square = 70.8 % | I-square = 56.6 % |

**★** Only 1 study; n.a.: not applicable
